# How teleworking adoption is changing the labor market and workforce dynamics?

**DOI:** 10.1371/journal.pone.0299051

**Published:** 2024-03-19

**Authors:** Yousif Elsamani, Yuya Kajikawa

**Affiliations:** 1 Department of Innovation Science, School of Environment & Society, Tokyo Institute of Technology, Tokyo, Japan; 2 Institute for Future Initiatives, The University of Tokyo, Tokyo, Japan; Lorestan University of Medical Sciences, ISLAMIC REPUBLIC OF IRAN

## Abstract

This article investigates how teleworking adoption influenced the labor market and workforce dynamic using bibliometric methods to overview 86 years of teleworking research [1936–2022]. By grouping the retrieved articles available on the Web of Science (WOS) core collection database, we revealed a holistic and topical view of teleworking literature using clustering and visualization techniques. Our results reflect the situation where the adoption of teleworking in the last three years was accelerated by the pandemic and facilitated by innovation in remote work technologies. We discussed the factors influencing one’s decision to join the workforce or a specific company, besides the unintended consequences of the rapid adoption of teleworking. The study can aid organizations in developing adequate teleworking arrangements, enhancing employee outcomes, and improving retention rates. Furthermore, it can help policymakers design more effective policies to support employees, improve labor force participation rates, and improve societal well-being.

## Introduction

Teleworking, also known as remote work or telecommuting, has been a subject of interest in the academic and business communities in recent years [1, 2]. The concept of teleworking can be traced back to the 1960s when some companies began experimenting with remote work arrangements for certain employees; however, it was not until the 1990s that teleworking began to gain wider recognition and acceptance as a viable alternative to traditional office-based work [3, 4]. Moreover, with the onset of the COVID-19 pandemic, many companies have turned to telework to maintain business continuity while ensuring employee safety [5]. This shift has increased companies’ willingness to adopt teleworking as a more permanent solution for their workforce as recent surveys show that more employees are working from home and that more than 80% of company leaders were planning to allow employees to work remotely some of the time after the pandemic [6, 7].

Besides its debatable benefits for the economy, society, and environment [8, 9], the increasing adoption of teleworking can have significant consequences for employees and employers. Many studies have explored the effects of teleworking on various aspects of work, such as job satisfaction [10], productivity [11], communication [12], social isolation [13], and work-life balance [14]. A recent systematic review study argues that while teleworking offers benefits like improved service delivery, enhanced satisfaction, support for healthcare providers, and cost reduction, it also faces challenges such as a lack of facilities, technology acceptance issues, and diminished interactions [15]. The study highlights the necessity for targeted management policies in healthcare to optimize teleworking’s advantages and mitigate its drawbacks. Some experts predict that teleworking could lead to a more decentralized operation and significant autonomy for teleworkers [16], with workers living further away from their workplaces and potentially working for companies in different countries [17, 18]. Others suggest that teleworking could increase job competition and reduce job security as companies can more easily outsource work to cheaper labor markets [19]. Previous studies have examined the impact of teleworking on some aspects of work, highlighting its influence on employees and the consequences for employers. However, they failed to adequately address the impact of the rapid adoption of teleworking on the labor market and workforce dynamics.

Since the literature on teleworking is enormous, one approach to gaining insight into the impact of teleworking on labor force dynamics is through bibliometric analysis. Bibliometric analysis is helpful when dealing with a large number of publications. It has been utilized to review topics and construct frameworks such as the relation between employees’ well-being and innovativeness [20], and female labor supply [21]. Thus, by examining the characteristics of teleworking literature before, during, and after the pandemic, bibliometric analysis can provide a comprehensive overview of the field, provide insights regarding the changes in workforce dynamics, help to identify gaps in knowledge, and highlight areas for future research.

Different expressions are used synonymously with teleworking (e.g., telecommuting, working from home (WFH), and remote working) [4]; thus, any attempt to review the literature on teleworking should consider other expressions used to refer to it. Researchers have made some effort to review studies on teleworking using bibliometric methods. For example, Herrera et al. reviewed the articles on the relationship between employees, family, and company using three keywords ("telework*," "e-working," or "electronic working") to retrieve articles from the Web of Science (WOS) database [22]. Teiusan and Deaconu attempted to map the research trends of teleworking using the same database, retrieving the articles published between 1975 and 2022 [23]. They used only one query to retrieve the articles ("teleworking"), retrieving 1,328 scientific publications. Other studies focused on the COVID-19 pandemic period. For example, Febriani and Churiyah utilized Scopus database to retrieve articles published between 2020 and 2022 using more comprehensive keywords: ("work from home," "telecommuting," "teleworking," and "remote work. “) ended up evaluating only 40 articles [24]. More recently, Tereza et al. presented a theoretical framework for remote work adaptation at personal, organizational, and governmental levels identifying trust, communication, and leadership as the most critical concepts in remote working research. However, the study is limited to using only WOS-indexed publications in the "Management" & "Business" category [25].

Previous teleworking and remote work bibliometric studies have provided valuable insights into the field. However, to our knowledge, studies have yet to comprehensively examine the teleworking literature using bibliometric analysis with a specific focus on the labor market and workforce dynamics. Therefore, our object was to review the existing research on teleworking, utilizing all available data on the Web of Science (WOS) database using all the common synonymous of teleworking. We topically mapped the teleworking literature highlighting the most critical quantitative data (such as research trends, most influential journals, and top authors). In doing so, we framed and discussed how the accelerated adoption of teleworking in the last three years has impacted labor market and workforce dynamics. This study can help organizations in their efforts to regulate and develop an effective teleworking arrangement, find more effective ways to enhance their employees’ outcomes and improve their retention rates. Furthermore, it can aid academic researchers in developing new theories and identifying teleworking-related topics that require further investigation. It can also help policymakers design more effective policies to support employees, improve labor force participation rates, and improve societal well-being.

## Data and methods

### Data

We downloaded the articles’ bibliographic data from the Web of Science (WOS) core collection database on January 24, 2023. We used all the common synonyms of teleworking to conduct our search: "telecommut*" OR "tele-commut*" OR "work*from home") OR "tele*work*") OR "tele-work*" OR "remote work*". The asterisk is used to include articles that use different spelling or combinations of words to refer to teleworking. For example, " telecommut*" will include word combinations such as telecommute, and telecommuting. We included all document types and downloaded the document that has one of the search queries either in the title, abstract or its keywords list. A total of 4,876 documents included at least one of the queries indicated above in either the title, abstract, or keywords list. Unlike other data sources, such as Scopus, data downloaded from WOS is easy and ready to use for bibliometric studies and is considered reliable for citation network analysis and other data mining studies [26]. In addition to academic articles, we used the same keywords to retrieve the patents data related to teleworking technologies from Derwent Innovation database. A total of 1,205 patents included at least one of the queries. We analyzed the data to investigate its trends and to understand how the fast adoption of teleworking during and after COVID-19 may have impacted the innovation in teleworking technology.

### Methods

Using bibliometric analysis and data mining techniques, we analyzed academic articles downloaded from the Web of Science. Each scientific paper was categorized into clusters centered around specific topics. This categorization involved creating connections between articles (represented as nodes) in the dataset and those referenced within them, employing a direct citation approach. The direct citation method effectively identified research topics across a broad spectrum of publications [27]. To refine our clusters and ensure relevancy, we included only strongly connected articles in our analysis, excluding weakly connected or disconnected ones based on predefined inclusion and exclusion criteria. The Louvain modularity maximization algorithm [28] was applied to form these clusters. Modularity, calculated using Eq 1, measures the density of connections within a cluster, with a higher value indicating a denser network of related articles.

$$Q=\sum_{s=1}^{M}\left[\frac{l_{s}}{l}-{\left(\frac{d_{s}}{2l}\right)}^{2}\right]$$ (1)

Where *M* is the number of clusters and *l*_*s*_ and *d*_*s*_ represent the number of links and the sum of the degrees of nodes within cluster *s*, respectively. The algorithm optimizes modularity in all nodes to form small communities that are then combined to form a single node. The best clusters are produced by this iterative process, which automatically determines the ideal number of groups or clusters.

**Fig 1:**
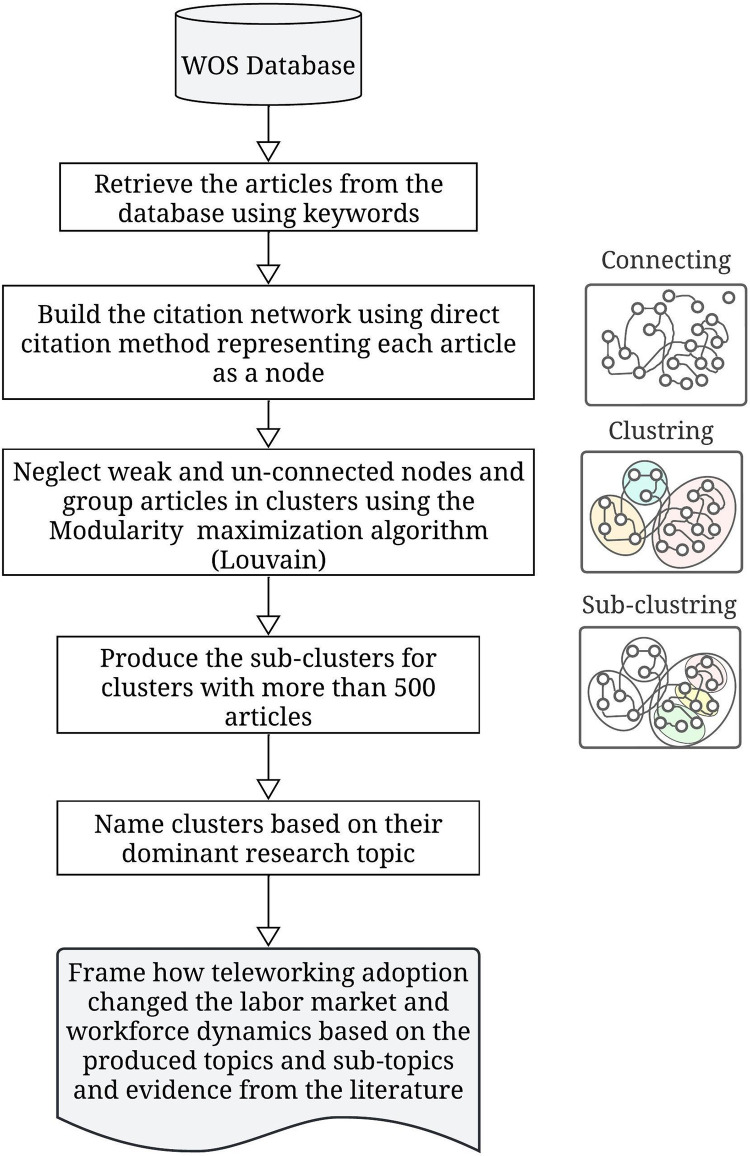
Overall summary of the steps and methods used to conduct this study.

The clusters consist of publications that reference each other, and since articles typically do not cite irrelevant works, we assume that they discuss similar or related topics. For ease of visualization, we assigned each cluster a distinct color [29]. To determine the primary topic of a cluster, we examined the most frequently cited articles, as well as top publishers, authors, and keywords. The same approach was used to generate sub-clusters within a given cluster, considering only articles within that cluster. According to earlier studies, the resolution limit problem may exist when the modularity optimization algorithm does not correctly identify smaller sub-clusters. However, it is more common in modules with a small number of internal links, less than √(2*L*), where *L* is the total number of links in the network [30]. The proposed framework is constructed by arranging all the topics, sub-topics, and factors produced through our analysis into four main parts: society, workforce supply, workforce demand, and unintended consequences. The dynamic between these 4 parts is discussed based on the evidence from the studies within our database. The overall steps are illustrated in Fig 1.

To understand how the articles within our dataset are compared, in terms of citation number, to articles outside the dataset, we computed the average number of citations received by the articles within each cluster (or sub-cluster) from other articles within the same cluster (referred to as *TC*_*c*_), from articles in the dataset that are considered as nodes (referred to as *TC*_*in*_), and from articles indexed in the WOS database (referred to as *TC*_*all*_). The ratios: *TC*_*c*_/*TC*_*in*_ and *TC*_*in*_/*TC*_*all*_ can be compared and plotted to show whether a particular cluster or sub-cluster received more citations from articles in that cluster, articles in other clusters, or articles outside our dataset (i.e., WOS database). In this study, we refer to this analysis as “citation relativity analysis.” If the ratio *TC*_*in*_/*TC*_*all*_ for a particular cluster (or sub-cluster) is relatively small this will indicate that the articles received more citations from articles outside of our dataset (i.e., articles not discussing teleworking); and similarly, small *TC*_*c*_/*TC*_*in*_ will indicate that the articles are cited more by articles outside the cluster (i.e., articles discussing topics different from the topic of the cluster under consideration) [31].

## Results

**Fig 2:**
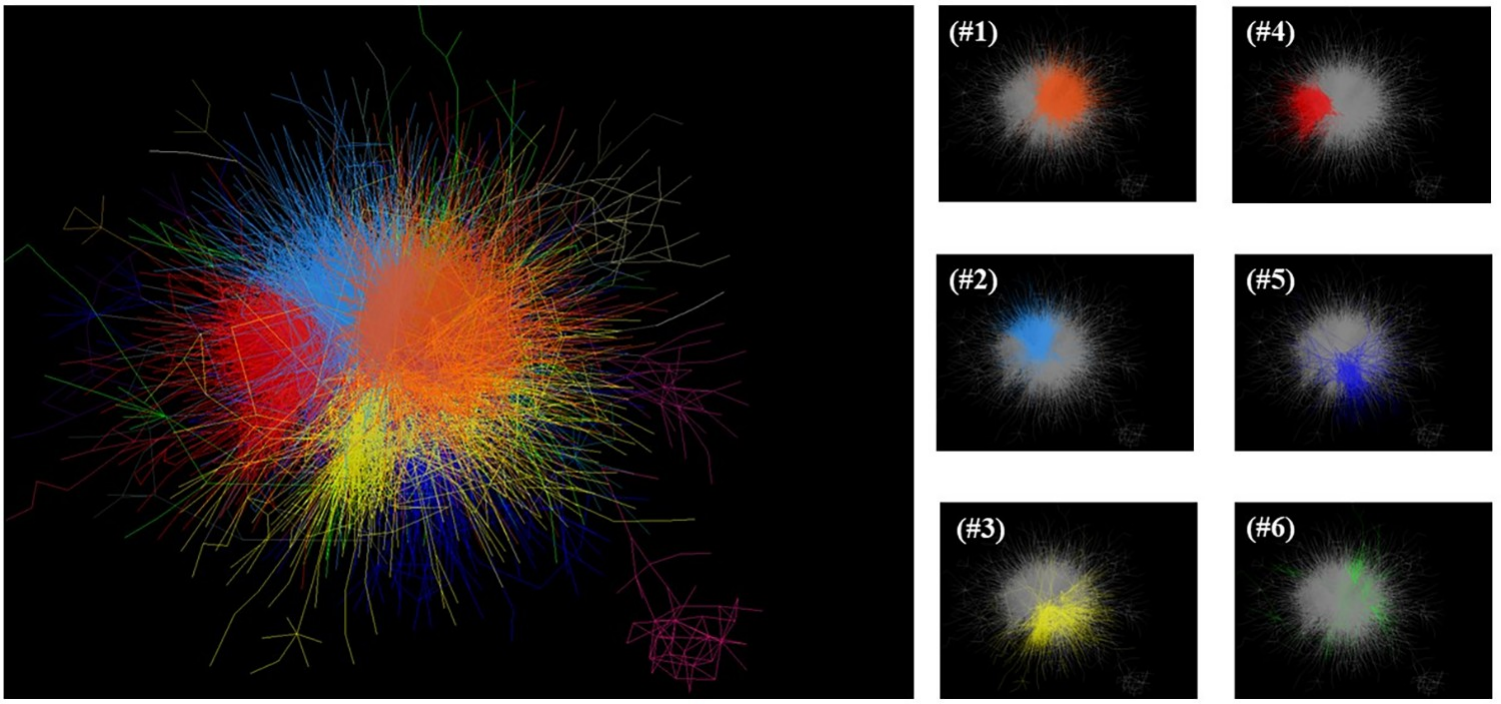
Clusters of teleworking research developed using direct citation network analysis and clustering methods. A total of 24 clusters were created and the figure shows the top 6 clusters.

**Fig 3:**
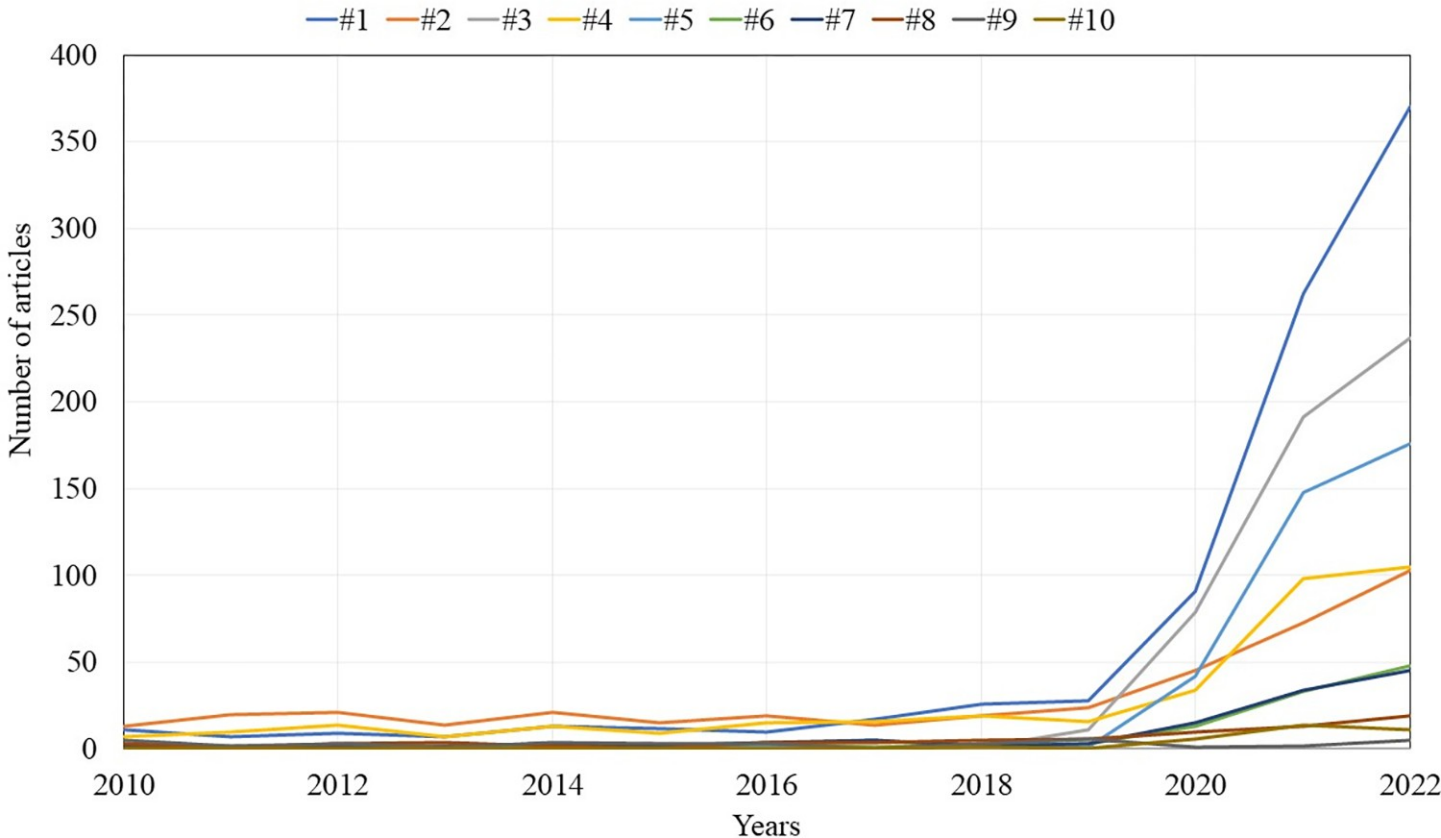
Trends of the top 10 clusters between 2010 and 2022.

The analysis resulted in 24 clusters and 25,479 links between 3,990 articles chosen as nodes. Fig 2 shows the overall network developed by the clustering and visualization techniques along with the top 6 largest clusters (in size). The top ten largest clusters represent 96.2% of the total articles chosen as nodes where the top 4 clusters contains more than 500 articles and the smallest one (cluster #10) contains only 37 articles. Table 1 shows the dominant topic, number of articles, average publication year, top three journals (in number of articles), most cited articles, and top three authors (in number of articles); and cluster trend for each one of the top ten clusters is shown in Fig 3. The average publication year of the articles chosen as nodes is 2017.4 and cluster 3 being the youngest cluster.

**Table 1:** The top 10 clusters are named using the dominant theme with the most important quantitative data (number of articles, average publication year, top 3 journals, most cited articles, and top 3 authors.

ID	Cluster’s Name	Articles (%)	Year (ave)	Top 3 Journals	Most Cited Articles	Top 3 Authors
#1	Teleworking consequences	1,100 (27.6%)	2019.6	Frontiers in Psychology International Journal of Environmental Research and Public Health (IJERPH) Sustainability	[4, 32, 33]	Golden, TD Toscano, F Allen, TD
#2	Teleworking benefits and pitfalls	705 (17.7%)	2011.5	New Technology, Work and Employment Sustainability Personnel Review	[18, 34, 35]	Tietze, S Higa, K Shin, B
#3	COVID-19	603 (15.1%)	2021	PLOS ONE IJERPH Gender, Work & Organization	[36–38]	Tateishi, S Tsuji, M Fujino, Y
#4	Travel, transportation, and traffic	543 (13.6%)	2014.1	Transportation Research Part A: Policy and Practice Transportation Transportation Research Record	[39–41]	Mokhtarian, PL Hensher, DA Beck, MJ
#5	Employee Well-being	433 (10.9%)	2020.8	IJERPH Work Sustainability	[42–44]	Nagata, T Fujino, Y Ikegami, K
#6	Software professionals	145 (3.6%)	2018.7	Human–Computer Interaction Economic and Political Weekly Lecture Notes in Business Information Processing	[45–47]	Moe, NB Smite, D Yang, LQ
#7	Cybersecurity	133 (3.3%)	2019.5	Sustainability Basel IJERPH Amfiteatru Economic	[5, 48, 49]	Foth, M Hearn, G Prosser, T
#8	Government and public organizations	96 (2.4%)	2016.8	Review of Public Personnel Administration Public Personnel Management Sustainability	[50–52]	Caillier, JG Kwon, M Charbonneau, E
#9	Work Home Interaction	43 (1.1%)	2014.1	Journal of Psychology in Africa South African Journal of Economic and Management Sciences Medycyna Pracy	[53–55]	Demerouti, E Moreno-Jimenez, B Bakker, AB
#10	Mental health and psychotherapy	37 (0.9%)	2020.2	British Journal of Psychotherapy Counselling and Psychotherapy Research Journal of Psychiatric and Mental Health Nursing	[56–58]	Dalton-Locke, C San Juan, NV Johnson, S
#11	Others	47 (3.8%)	-	-		-

**Fig 4:**
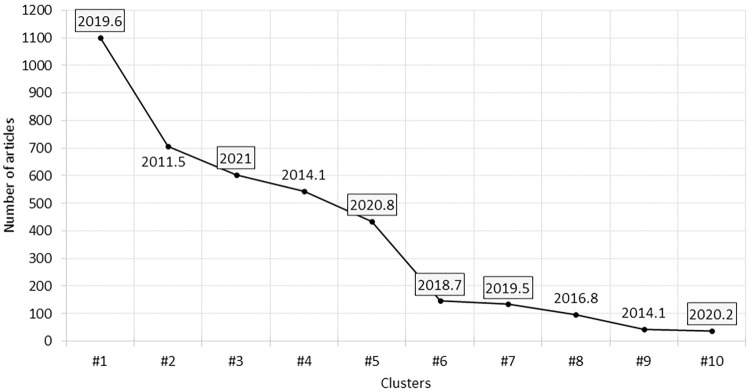
Average publication year of the top 10 clusters. Six clusters (highlighted) are emerging clusters that had an average publication year within the last four years (i.e., after 2018).

Fig 4 shows the average publication year of the top 10 clusters, revealing that six clusters have an average publication year after 2018 (i.e., within the last four years). The number of articles in these clusters represents more than 61% of the articles chosen as nodes. Teleworking as a research field can be considered an emerging field of study (the overall average publication year is 2017.4); with nine clusters of the top 10 clusters having an average publication year within the last 9 years. The youngest cluster (i.e., with the most recent average publication year) is cluster #3 which mainly discusses teleworking (i.e., working from home) during the COVID-19 pandemic. Cluster # 5 is the second youngest cluster that discusses well-being related topics in teleworking context followed by cluster #10 which also discusses mental health and psychotherapy. This can be an indicator that well-being and mental health issues are one of the main concerns in working from home during the pandemic which lead teleworking researchers, especially during the pandemic, to investigate its impact on well-being regarding employees and their families.

Cluster #1 is the largest, accounting for 27.6% of the total publications, with Teleworking consequences being the most prevalent topic. The most cited paper is a meta-analysis study that focuses on the psychological mediators of teleworking on individuals [32]. The study analyzed 46 studies, involving 12,883 employees, arguing that autonomy mediated the beneficial consequences of teleworking (e.g., job satisfaction and performance) and that high-intensity telecommuting has both beneficial (on work-family conflict) and harmful (relationships with coworkers) effects. The second most cited article investigated teleworking effectiveness after reviewing teleworking definition including its other synonymous [4]. Moreover, one of the major focuses of researchers in this cluster is the impact of teleworking on employees where it was to have a significant impact on their well-being [59], job satisfaction [10], stress levels [60], work-family conflict [61], and work-life balance [62], and other aspects related performance and turnover intentions [13]. The topics discussed in this cluster can be considered emerging topics (with average publication year of 2019.6), discussing teleworking outcomes that was imposed by COVID-19 pandemic.

Cluster #2 is mainly discussing teleworking benefits and pitfalls representing 17.7% of the total articles chosen as nodes. The cluster discusses teleworking as a new way of working and have an average publication year of 2011. The most cited article reviewed the literature on telework and found that work-related factors such as managers’ willingness are most predictive of which employees will telework and finds clear evidence that telework increases job satisfaction and productivity [34]. Regarding, the perceptions of teleworking among professionals and managers, Baruch explored its advantages and disadvantages through interviews conducted with 62 teleworkers [35]. The study identified the people who may be best fit to teleworking (self-discipline being the most important attribute) and that having a home office, to be able to distinguish between work and facets of life, can be crucial.

Cluster #3 represents 15.1% of the total publications, with COVID-19 being the most prevalent topic and average publication year of 2021 representing the youngest cluster (Fig 4). During the pandemic enterprises have adopted telecommuting as a strategy to secure the safety of their workforce and to sustain the flow of economic operations [5]. The most cited articles discuss how we can achieve effective remote working during the COVID-19 pandemic and identified four main challenges: work-home interference, ineffective communication, procrastination, and loneliness [33]. They argue that virtual work factors such as social support, job autonomy, monitoring, and workload affected how teleworkers may experience these challenges; with one major individual difference factor: self-discipline. The cluster also discusses topics such as the characterization of working from home [63], working population [64] and the impact of working from home, during COVID-19 pandemic, and technology use on teleworkers’ well-being [65].

**Fig 5:**
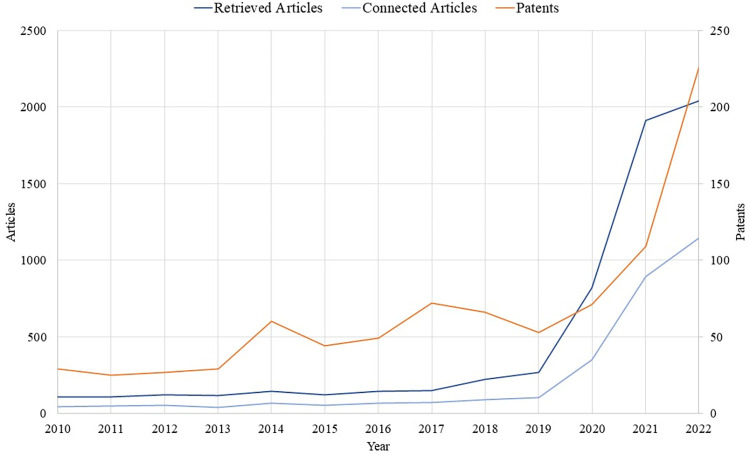
Number of articles (retrieved and connected) and number of patent applications between 2010 and 2022.

In 2020 (during the COVID-19 pandemic), there was a noticeable increase in the number of articles discussing teleworking, which was followed by an even more significant increase in 2021 and 2022. This is also true for the number of patents related to teleworking technology (Fig 5). The patents data were downloaded from Derwent Innovation database using the same keywords which were utilized to download the articles data from WOS. The retrieved articles, in Fig 5, refer to the articles downloaded from WOS before conducting the citation network analysis, whereas the cited articles represent the most cited and connected articles within the network. We believe that due to the pandemic-imposed adoption of teleworking, responding to social distancing regulations in many countries, researchers put more effort to discuss and investigate teleworking issues. Until the day of data retrieval, the articles published between 2020 and 2022 represent more than 60% of the total literature published on teleworking since 1976. The articles were published in 1,740 different journals, with the International Journal of Environmental Research and Public Health (IJERPH), Sustainability, Frontiers in Psychology, and New Technology, Work and Employment receiving the most publications, respectively. Among the top 10 journals, there were 3 journals focused on transportation (TRANSPORTATION RESEARCH PART A POLICY AND PRACTICE, TRANSPORTATION, and TRANSPORTATION RESEARCH RECORD) which can be a clear indication of the significant impact on transportation due to teleworking adoption.

The main topic of cluster #4 is travel and transportation, focusing on topics such as the approaches used to estimate the impacts of telecommuting on travel [39], and its impact on energy use and climate [40]. Hook et al. concluded that twenty-six of the reviewed studies (total of 39 articles) suggest that teleworking reduces energy use with the main sources of saving being the reduced distance travelled and lower office energy consumption. However, the article argues that rigorous studies with wider impact range show smaller savings and emphasizes the uncertainties about the actual or potential benefits indicating that, in many circumstances, economy-wide energy savings could be modest, non-existent, or even negative. Other studies focuses on the methodological issues regarding the impact of teleworking on energy saving [66] and air quality [41]. Another main issues discussed in this cluster are factors that drives or constrain telecommuting [67], where attitudinal measures were found to be more important than sociodemographic characteristics; and the implications for transportation networks in urban territories where also introduced [68].

Cluster #5 discusses employees’ well-being. For example, the most cited article investigated how working from home during COVID-19 pandemic impacted the physical and mental well-being of office workstation users [42], where it found that after working from home there was a decrease in the overall physical and mental well-being owing that to some factors such as physical exercise, food intake, and communication with coworkers. Other main issue discussed in this cluster includes musculoskeletal problems [69], sleep [70] and lifestyle [71]. Physical well-being was major concerned during the lockdown since employees sitting time increase during working from home days [72] along with decrease in physical functioning [71].

Other clusters have the following characteristics. Cluster #6 focused on software professionals and how, for example, pandemic affected software developers and the ways organizations can provide support to offset the negative effects on well-being and productivity [46]. Cluster #7 discussed the cybersecurity implications of the rapid adoption of teleworking. During the pandemic, organizations needed to protect their information systems against unauthorized access were organization have been targeted, especially healthcare organizations, making cybersecurity a major concern that need an urgent attention [73]. Cluster #8 focuses on government and public organizations, investigating issues such as the work motivations among federal government agencies [51], leave intentions report compared to non-teleworkers [50], and the impact of decoupling of telework on job satisfaction [74]. The average publication year of this cluster is 2016 which could indicate that these topics were not revisited during the pandemic.

Cluster #9 discusses home-work interaction discussing issues such as work interference and work-family conflict. The most cited article looked at how home-work interference may influence performance arguing that the need for recovery and home-work interference could negatively affect concentration and lead to decreased performance over time [53]. The Survey Work-Home Interaction Nijmegen (SWING) is utilized widely among the studies within this cluster where it was translated and adopted in many countries such as Spain [75], and Netherlands and South Africa [76]. The final cluster (#10), discusses mental health and psychotherapy. COVID-19 impacted people’s mental health and potentially burden care system and mantal health service users [56]. The study overviewed the concerns and experiences of UK mental health care staff working in the early pandemic and concluded by suggesting combining infection control and a therapeutic environment in hospitals and effective telehealth implementation in the community.

**Fig 6:**
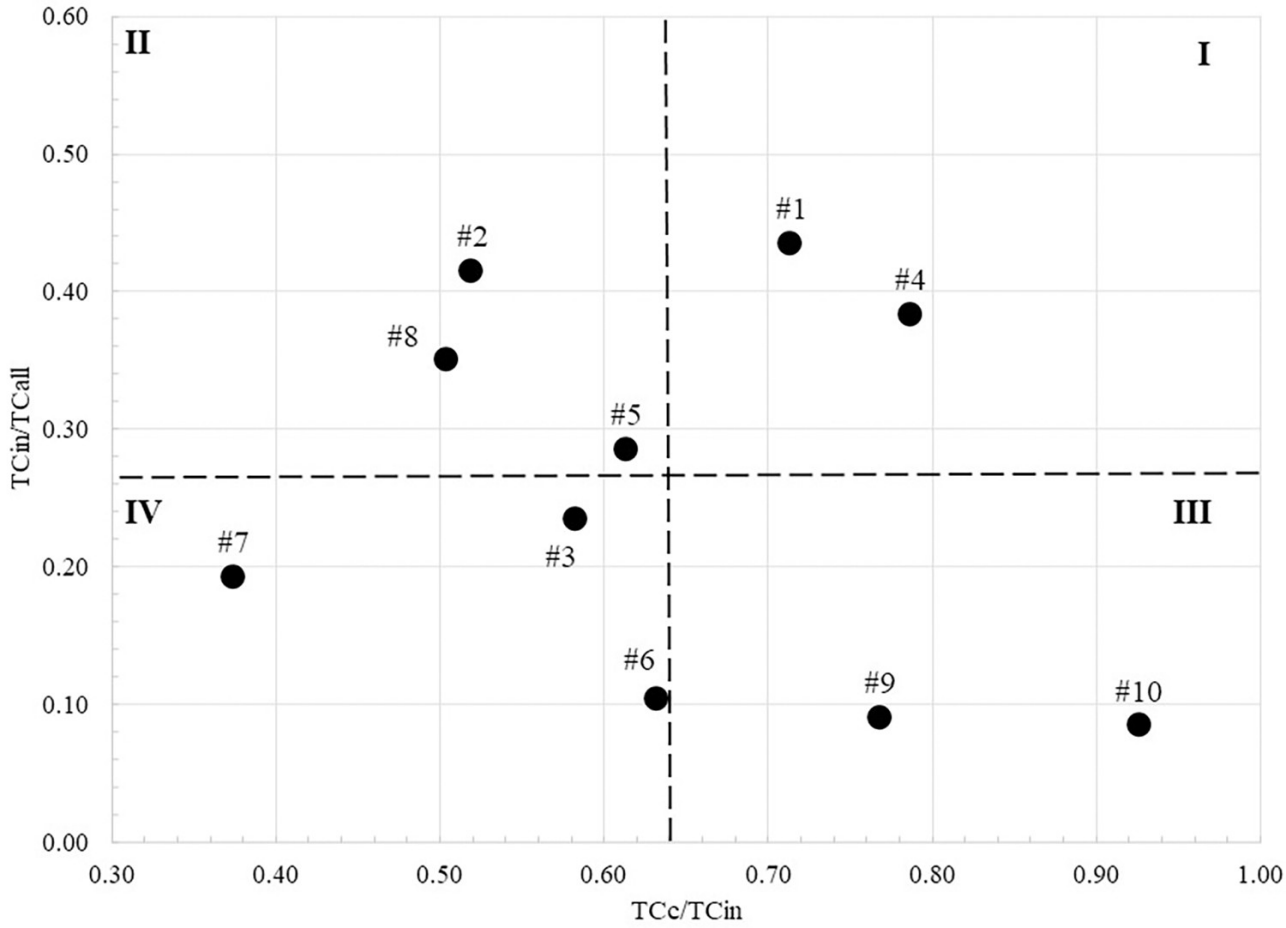
Citation relativity illustration of the top 10 clusters. The dashed lines represent the average values of ratios. (I) Topic specific; (II) domain specific; (III) specific & global; (IV) global.

As explained in the Data and Method section, we performed the citation relativity analysis on the top 10 produced clusters. Table 2 depicts the results obtained by calculating the variables needed for the analysis. The column “Links” in the tables represents the number of connections created between the articles in each cluster. Fig 6 illustrates the result of the citation relativity analysis. Cluster 1 (Teleworking consequences) and cluster 4 (Travel, transportation and traffic), compared to the other clusters, have relatively large *TC*_*c*_/*TC*_*in*_ and *TC*_*in*_/*TC*_*all*_; which indicate that the issues discussed in these clusters are topic specific (articles are mostly cited by articles belong to the same cluster and mostly cited within our dataset). On the other hand, cluster 7 (Cybersecurity), cluster 6 (Software professionals), and cluster 3 (COVID-19) can be considered global, which means that the articles here are more cited by articles outside the cluster and at the same time by articles outside our dataset. This makes sense because we can anticipate topic such as COVID-19 and Cybersecurity are discussed by other research fields from many different prospectives whereas teleworking consequences, and travel and transportation are more associated with telecommuting.

**Table 2:** Average number of citations for the top 10 clusters.

ID	Cluster’s Name	Articles	Links	Average Citation	*TC*_*C*_	*TC*_*in*_	*TC*_*all*_
1	Teleworking consequences	1,100	6,454	5.9	5.87	8.22	18.93
2	Teleworking benefits and pitfalls	705	3,395	4.8	4.82	9.27	22.38
3	COVID-19	603	1,226	2.0	2.03	3.49	14.87
4	Travel, transportation, and traffic	543	3,522	6.5	6.49	8.25	21.53
5	Employee Well-being	433	963	2.2	2.22	3.62	12.72
6	Software professionals	145	211	1.5	1.46	2.30	22.25
7	Cybersecurity	133	188	1.4	1.41	3.78	19.65
8	Government and public organizations	96	297	3.1	3.09	6.14	17.49
9	Work Home Interaction	43	53	1.2	1.23	1.60	17.72
10	Mental health and psychotherapy	37	50	1.4	1.35	1.46	17.27

**Fig 7:**
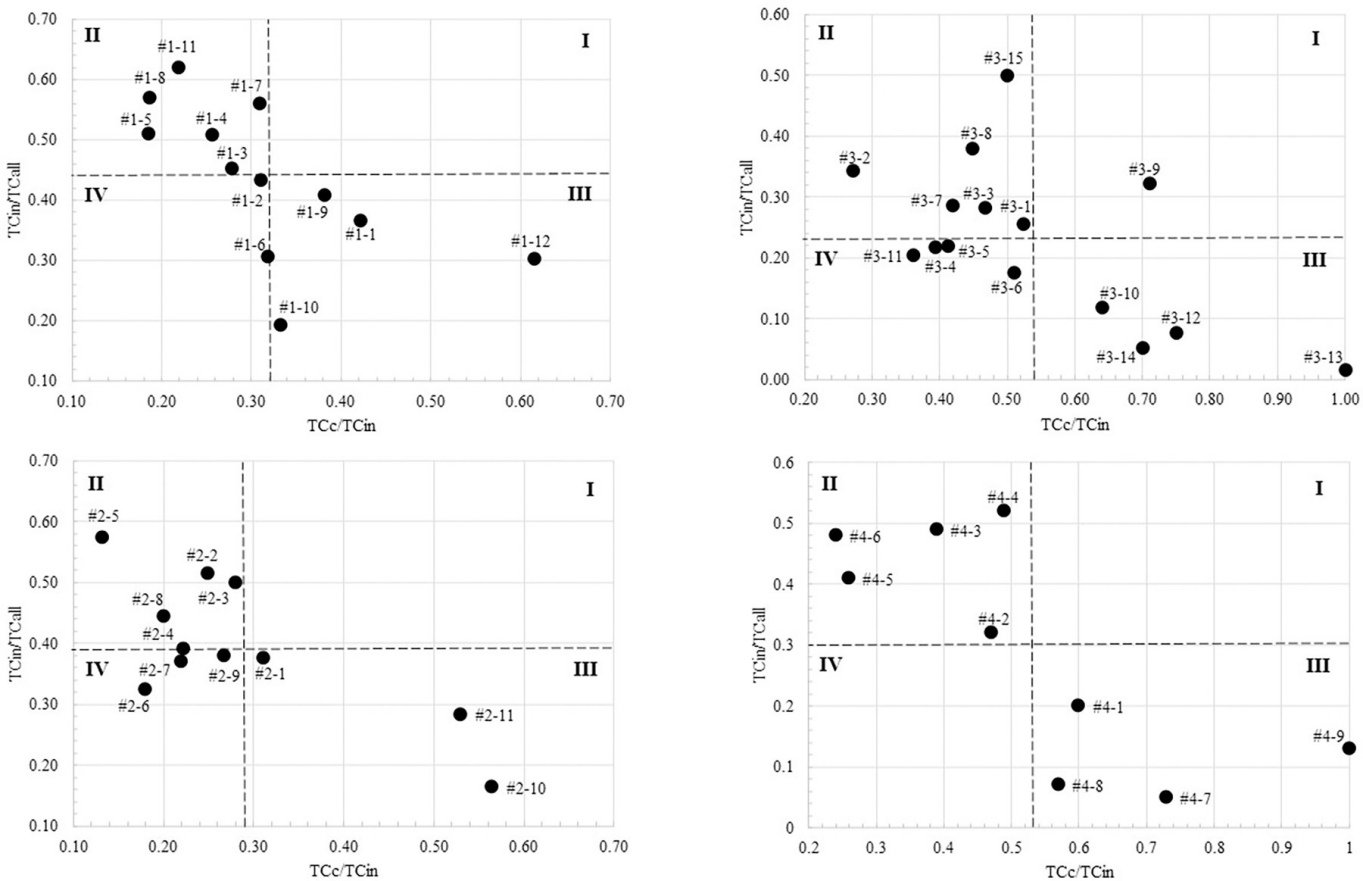
Citation relatively illustration of the 2nd level clusters of the top 4 clusters. The dashed lines represent the average values of ratios. (I) Topic specific; (II) domain specific; (III) specific & global; (IV) global.

The top 4 clusters, in size, contain more than 500 articles; thus, we produced their sub-cluster to have a better understanding of the sub-topics investigated at each cluster (Tables 3–6). The citation relativity analysis of the produced sub-clusters was also conducted and illustrated in Fig 7.

**Table 3:** Average number of citations for the 2nd level sub-clusters of clusters #1.

ID	Cluster name	Articles	Year (ave)	Links	Average Citation	*TC*_*C*_	*TC*_*in*_	*TC*_*all*_
#1	Teleworking consequences	1,100	2019.6	6,454	2.4	2.38	5.64	15.44
#1–1	Pandemic-imposed teleworking	224	2021	534	3.8	3.78	12.15	28.14
#1–2	Work performance	162	2016.8	613	1.9	1.93	6.93	15.34
#1–3	Work-life balance	135	2020.5	261	2.7	2.66	10.32	20.31
#1–4	Communication and isolation	117	2018.1	311	2.1	2.10	11.29	22.16
#1–5	Employee Well-being	103	2019.1	216	2.2	2.24	7.02	23.01
#1–6	Work and home boundary	99	2020.7	222	2.2	2.19	7.06	12.59
#1–7	Work engagement	69	2021.2	151	2.1	2.12	11.32	19.91
#1–8	Job satisfaction	65	2019.2	138	1.4	1.44	3.78	9.27
#1–9	Family conflict	45	2020.4	65	1.3	1.29	3.87	20.03
#1–10	Management and leadership	38	2019	49	1.6	1.60	7.31	11.80
#1–11	Productivity	35	2021.2	56	1.0	1.00	1.63	5.38
#1–12	Innovativeness	8	2021.7	8	2.4	2.38	5.64	15.44

**Table 4:** Average number of citations for the 2nd level sub-clusters of clusters #2.

ID	Cluster name	Articles	Year (ave)	Links	Average Citation	*TC*_*C*_	*TC*_*in*_	*TC*_*all*_
#2	Teleworking benefits and pitfalls	705	2011.5	3,395	3.2	3.19	10.25	27.28
#2–1	Home-based working	118	2015.1	376	3.1	3.13	12.52	24.32
#2–2	Organizational control	101	2010.9	316	2.2	2.18	7.76	15.54
#2–3	Emergence of Telecommuting	96	2003.4	209	1.9	1.87	8.42	21.54
#2–4	Disability	78	2009.2	146	1.6	1.55	11.76	20.47
#2–5	Teleworking adoption	58	2015.2	90	2.0	1.98	11.04	34.05
#2–6	Teleworking evolution	57	2010.3	113	1.8	1.75	7.98	21.59
#2–7	Mobile teleworkers	56	2015.3	98	1.6	1.61	8.02	18.08
#2–8	Virtual workplace	49	2013.1	79	2.3	2.28	8.54	22.52
#2–9	Home-based entrepreneurship	46	2009.8	105	1.6	1.58	2.81	17.06
#2–10	Coworking	36	2020.3	57	0.9	0.90	1.70	6.00
#2–11	Public implications	10	2009.6	9	3.2	3.19	10.25	27.28

**Table 5:** Average number of citations for the 2nd level sub-clusters of clusters #3.

ID	Cluster name	Articles	Year (ave)	Links	Average Citation	*TC*_*C*_	*TC*_*in*_	*TC*_*all*_
#3	COVID-19	603	2021	1,226	2.20	2.20	4.19	16.44
#3–1	Teleworking compatibility	125	2021.5	275	1.45	1.45	5.32	15.53
#3–2	Alternative work arrangements (gig)	76	2020.8	110	2.29	2.29	4.89	17.39
#3–3	Gender	70	2020.3	160	1.08	1.08	2.73	12.59
#3–4	Lockdown and home offices	66	2021.4	71	1.35	1.35	3.25	14.82
#3–5	Adaptation	55	2021.1	74	1.32	1.32	2.58	14.70
#3–6	Female academics	50	2021.1	66	1.16	1.16	2.77	9.72
#3–7	Regional implications (real estate)	43	2021.3	50	1.08	1.08	2.40	6.32
#3–8	Individual habits and activities	25	2021.5	27	1.39	1.39	1.96	6.09
#3–9	Impact on libraries	23	2019.1	32	1.09	1.09	1.70	14.30
#3–10	Unpaid care	23	2021.4	25	1.00	1.00	2.76	13.59
#3–11	Social support (childcare)	17	2021.3	17	0.92	0.92	1.23	16.23
#3–12	Human mobility	13	2021.3	12	0.88	0.88	0.88	56.38
#3–13	Social contact	8	2020.5	7	1.00	1.00	1.43	27.71
#3–14	Digital transformation	7	2020.4	7	0.50	0.50	1.00	2.00
#3–15	Loneliness	2	2021.5	1	2.20	2.20	4.19	16.44

**Table 6:** Average number of citations for the 2nd level sub-clusters of clusters #4.

ID	Cluster name	Articles	Year (ave)	Links	Average Citation	*TC*_*C*_	*TC*_*in*_	*TC*_*all*_
#4	Travel, transportation, and traffic	543	2014.1	3,522	6.49	8.25	21.53	543
#4–1	Travel patterns and activities	116	2019.2	290	2.50	2.5	4.16	20.78
#4–2	Energy and emission	107	2016.2	298	2.79	2.79	5.96	18.51
#4–3	Telecommuting choice	91	2011.7	379	4.16	4.16	10.73	21.71
#4–4	Household travel	87	2014.8	521	5.99	5.99	12.1	23.31
#4–5	Telecommuting forecasting	68	2003.6	182	2.68	2.68	10.24	24.87
#4–6	Teleworkers characteristics	59	2014.9	145	2.46	2.46	10.41	21.63
#4–7	Traffic (Ruch hour)	7	2016	8	1.14	1.14	1.57	30
#4–8	Public transit	5	2017	4	0.80	0.8	1.4	20.2
#4–9	Counter urbanization	3	2021.7	2	0.67	0.67	0.67	5.33

## Discussion

The pandemic has rapidly accelerated the teleworking adoption however caught employees and organizations off guard since the adoption was not gradual and most companies did not have a teleworking program in place [77]. Our analysis shows that there has been a big increase in the number of articles discussing topics related to teleworking in the past three years; representing more than 60% of the total literature published on teleworking. Additionally, we can generally divide research clusters into two main groups: pre- and post- pandemic. In pre-pandemic literature, the analysis results indicate that researchers discussed teleworking as an emerging new way of working (cluster #2: Teleworking benefits and pitfalls), investigating issues such as the emergence and evolution of teleworking (cluster #2–3, and #2–6) and how organizations and employees are adapting when teleworking is implemented (cluster #2–5). Additionally, articles in cluster #2 investigated other working arrangements such as mobile teleworkers (cluster #2–7), virtual workplaces (cluster #2–8), and coworking (cluster #2–10). Regarding the articles published during and post-pandemic, COVID-19 was the central topic (Cluster #3: COVID-19), and researchers investigated the consequences of the rapid adoption of teleworking.

During the pandemic, employees continued to work and provide their services working from home; thus, organizations became more accepting and supportive of teleworking [78]. The rapid adoption of teleworking and the fact that organization have become more accepting to hire teleworking employees, as an adaptation strategy or out of necessity, has influenced the labor force dynamic (supply and demand) and led to unintended consequences that have some implications for all stakeholders: employees, organizations, and society [79, 80]. In this section we attempt to synthesize our analysis result to frame and discuss and the changes in labor market and workforce dynamics. As briefly mentioned in the methods section, we construct our framework by organizing the topics, sub-topics, and factors generated through citation network analysis into four main categories: (I) society, (II) workforce supply, (III) workforce demand, and (IV) unintended consequences as below.

### Society and supply-side factors of workforce

For the first category (Society), we included three main topics or issues discussed in the literature: Private life issues, Gender issues (cluster #3–3), and social support (cluster #3–11). Private life represents issues related to people’s personal life which includes well-being (cluster #5), work home interaction (cluster #9) mental health (cluster #10), work life balance (cluster #1–3), work and home boundary (cluster #1–6), and family conflict (cluster #1–9).

Individuals can join the labor force working as a regular employee in offices or on sites, or through teleworking compatible jobs (full time or hybrid) which can be more suitable for people with special needs (such as people with disability) or people with certain situations (i.e., working women and other work arrangements). During and post-pandemic labor supply pool has increased, and more people (with different needs and situations) have been able to join the labor force. For example, considering only people with disability, in October 2022, the participation rate reached 37.6% for women and 39.7% for men, increasing about 5% from April of the same year [81]. Since employees with disabilities are more likely than those without disabilities both to work primarily from home and to do any work at home, that may have been one of the reasons driving this increase [82]. Another driver of the increase in participation rate could be due to the post-pandemic surge in the number of newly founded businesses (startups), which saw a sharp rise in the number of applications for new companies from the second half of 2020 to May 2021 [83]. The increased participation rate through teleworking is fundamentally accelerated by COVID-19 pandemic and facilitated by the advancement of ICTs, particularly remote work technology, in public and private sectors [3].

The accelerated adoption of remote work may have also pushed organizations that are developing remote work technologies to invest more and find more innovative solutions for remote work issues, leading to a 100% increase in the share of new patent applications that advance teleworking technologies in the period between January to September of 2020 [84]. Additionally, our analysis showed that the sharp increase in the number of patents continued through 2021 and 2022 (Fig 5). Innovation in remote work and collaborative technologies may improve communication, which can be important for companies with remote teams, as teleworking technology facilitates collaboration and fosters a sense of connection among team members [85]. Additionally, new technologies, such as virtual reality [86] and metaverse platforms [87] are some examples of promising technologies that can enrich the communication experience between teleworkers.

Noticeably, well-being and its related constructs (such as mental health and work life balance) were dominant in the post-pandemic teleworking literature (commonly referred to as "working from home"). Well-being regarding oneself or family members (e.g., children) is critical since it can influence the individual’s decision to join the labor force [88, 89]. Using social exchange theory, Kelliher et al. argue that teleworking can lead to work intensification, where employees are trading flexibility for effort [90]. However, flexibility can increase parent-child interaction, especially for fathers working in dual-earner households [91]; and for working mothers, work flexibility can enhance the sense of balancing work and life and the perceived quality of life [92]. Although flexible work has been shown to increase women’s participation, Suri suggests that to improve and sustain this participation, we need to offer accessible and affordable childcare [93]. Moreover, the COVID-19 pandemic has presented challenges for various scholarly groups, including female academics (cluster #3–6). These challenges include struggles with maintaining mental health and difficulties achieving a satisfactory balance between work and home responsibilities [94]. These challenges are also faced by single women academics with no children, despite the narrative that they may enjoy a surge of productivity during the pandemic [95]. As argued by the authors, this assumption must be nuanced because living alone in the context of the pandemic presents its own set of circumstances and challenges that affect academic productivity and work-life balance. Freelancers and gig workers face similar challenges where their irregular working hours get prioritized over their domestic commitments, causing work-family conflict and eroding work-family boundaries [96]. Shevchuk et al. investigated freelancers’ challenges (Russian language internet translators) and found that they experience adverse socio-economic outcomes in several dimensions: reduction in earnings, decreased job satisfaction, and expressing intentions to change their current employment situation [97]. Another prevalent type of remote working is gig working [98], but since we did not include gig worker-related keywords in our search, we only found a small number of publications. Among our dataset (articles chosen as nodes), one study investigated how organizations could mitigate misconduct among gig and remote workers, suggesting that it is common in such contexts and can be mitigated by communicating organizational values and a credible threat of monitoring [99]. Another alternative to the traditional home office can be coworking spaces. Because it has implications for social interactions and health-related factors, it can be the preferred work arrangement compared to the home office [100]. Beside the communal aspects of coworking, we may also need to investigate the inputs, outputs, and outcomes of coworking using quantitative approaches. Robelski et al. found that coworkers reported enhanced levels of innovation, despite innovation not being an explicit motivation for choosing coworking spaces [101]. Beyond working from home or remote offices, Hislop et al. noted that the literature on teleworking needs to examine the experiences of mobile teleworkers (cluster #2–7), who conduct their business away from their homes and offices, developing a theoretical framework addressing examples related to their work-life balance [102].

The decision to join a particular company can be influenced by the availability of options to work from home, and organizations are attempting to attract talent by offering this option. Darby et al. uncovered that, by monitoring 47 million job adverts, the language has changed to explicitly mention opportunities for working from home and that the characteristics of positions have also changed [103]. Another factor that influences employee decisions is career development. Since central location (i.e., company offices) visibility is considered critical for outstanding performance evaluation [104], teleworkers (specially full-remote employees) may be concerned about their career development [105] and mentoring [106]. Such factors can influence the employee’s decision to join and stay at a company [107]. On the other hand, to be considered for positions that provide a teleworking option, employees need to develop their digital skills since the need for digital literacy is growing [108].

### Demand-side factors of workforce

On the demand side, or organizational side, our analysis showed that management and leadership (cluster #1–10), organizational control (cluster #2–2), and support are vital and can impact teleworkers’ output by influencing their productivity, performance, work engagement, and innovativeness. Employee well-being and communication between teleworkers are also critical and can affect their sense of belongingness, collaboration, and coordination, which can influence the mentioned outputs [20].

Work technology issues such as organization control and surveillance are considered critical [109]. Organizations aspire to influence their employees to act according to the company’s rules, values, and vision; thus, for teleworking employees, how organizational control is implied and implemented can be vital [110]. Chatterjee et al. suggest that organization policy and top management support play crucial roles in implementing remote work policies and that remote work flexibility significantly enhances organizational performance [111]. Organizations may rely on the supervisor’s discretion regarding implementing their policies. However, to effectively manage teleworking, research suggests that supervisors should focus on information sharing rather than monitoring, encourage clear establishment of boundaries [112], and put more emphasis on output controls [113]. However, telecommuters who are encouraged to create boundaries between work and family are less likely to help colleagues in crunch times or after hours, which may increase non-telecommuter’s workload and work-family conflict [112]. Establishing a shared "culture of control" is essential to ensure coherence and consistency in the attitudes and behaviors of team members [114]. In complex projects, standard and straightforward procedures are critical for establishing a shared culture; and the leaders’ capabilities to guarantee the teleworkers’ alignment and foster collaboration and creativity are even more critical [115, 116]. The discussed factors (i.e., teleworking management, leadership, and organizational control) can have a direct and indirect influence on employees’ outcomes such as productivity, performance, and work engagement.

One of the earliest studies in our dataset to investigate productivity in a teleworking context is by Jeffrey Hill et al., where they suggest that telework can yield greater productivity, higher morale, increased flexibility, and longer work hours [117]. Other researchers argue that the impact on productivity can be linked to other factors such as residential environment and personality traits [118], the use of video conference meetings [119], and perceived autonomy (measured by physiologic effort) [120]. A pre-pandemic study (2013) investigated the effect of working from home four days a week on employees’ productivity in a Chinese company call center. They found that it increases productivity by 13% and that their attrition rates are improved, indicating an elevated level of satisfaction and well-being [37]. According to the employees, the primary reason for this improvement is that they do not have to commute, but most importantly, it is quieter at home. This study was followed up by another in 2021, focusing this time on employees working in teams and doing more creative work (600 participants who work 2 days a week). This study has four key results: the employees were happier being able to work two days a week from home (confirmed by surveys and in attrition rates), teleworking changed the structure of hours (reduced workday hours to be compensated on the weekends), changed the way they communicate with their co-workers (more messaging) even in the office days, and there was a trivial increase in productivity [121].

The impact of teleworking on organizations, teams, and individual performance was also one of the primary focuses. Sanchez et al., in a pre-pandemic study (2007), argue that firm performance is positively related to the use of teleworking and flextime since such firms have more employees involved in job design and planning and are more intensively managed by results [122]. This is also true for small businesses, where working from home has helped them perform better during the pandemic [123]. Although researchers argue that working from home improves performance [4, 124], others have some concerns regarding its negative consequences since it could lead to social and professional isolation that restricts knowledge sharing [105] and increases labor intensity [90]. On the individual and team level, a meta-analysis study suggests that teleworking positively impacts employees’ performance, both supervisor-rated and subjective [32]. Although, when accounting for the co-worker effects, can negatively impact employees’ performance, highlighting the impact employees have on each other and how they take advantage of each other’s expertise [125]. Full-time telework might not be the optimal situation for organizational performance, whereas partial home working has been considered an optimal solution for increasing organizational performance, social and professional relationships, learning and personal development, and the overall level of work motivation [126].

Since work engagement (cluster #1–7) is one of the aspects that can be influenced by teleworking, organizations aspire to keep employees engaged by reducing job demands and increasing resources which can be achieved by reducing work pressure and role conflict and increasing autonomy [127]. In a more recent study, Wang, H et al. investigated the role of family-supportive leadership in the relationship between home-based telework and work engagement, arguing that family-supportive leadership weakens the negative effect of home-based telework on work engagement [128]. Therefore, organizations need to cultivate family supportive leadership to ensure employees remain engaged while working from home.

Communication between teleworking employees (cluster #1–4) is one of the main topics in teleworking literature, where employees utilized internet communication technologies (ICTs)—for the first time during COVID-19 pandemic in the case of workers with no prior teleworking experience—to interact and share knowledge. The impact of professional isolation is increased by the time spent teleworking, whereas more face-to-face interactions and access to communication-enhancing technology tend to decrease its effects [13]. Cooper CD et al., argue that how much an organization values developmental activities and how much a telecommuter misses them determines how isolated they feel. These activities include interpersonal networking, informal learning, and mentoring. Telecommuting is less likely to impede the professional development of public sector employees because they value these activities less than their private sector counterparts do [129]. Since teleworkers communicate more using phones and video calls, Smith et al. investigated how satisfaction with communication channels can influence the relationship between employee personality and job satisfaction, concluding that extraversion, openness, agreeableness, and conscientiousness are positively correlated with job satisfaction. On job satisfaction, significant moderating effects were discovered in the relationships between openness and phone and video communication, as well as agreeableness and phone communication [12].

Researchers investigated how effective virtual communication is and how they may impact innovativeness (cluster #1–12). Creativity is the idea generation stage of innovativeness (individual innovation), which is a multi-stage process that starts with problem recognition, idea generation (creativity), idea promotion, and finally, implementation [130]. Thus, a negative impact on one of these stages will impact innovation on an individual level and, as a result, on an organizational level. The frequency of face-to-face interactions between employees, measured using sociometric data, is associated with creativity [131]. When it is replaced with videoconferencing, it inhibits the production of creative ideas, but selecting which idea to adopt can be more effective [132]. In software development, time pressure and autonomy can mediate the positive correlation between telework and creativity among professional employees [133]. Other research suggests that creativity can be predicted by the employee psychological profile, where those with a “solitary” profile perceived themselves as less creative and produced objectively fewer ideas than individuals with an “affiliative” profile [134]. Another way of looking at this issue is the association between commuting and employee innovation performance. Commuting is found to harmful to inventor’s productivity, and every 10 km increase in distance is associated with a 5% decrease in patents per inventor-firm pair per year and an even 7% decrease in patent quality [135]. Weiwei Huo et al. argue that the impact on employees’ innovative behavior can differ depending on whether telecommuting is voluntary. They investigated that through co-worker emotional support and explored the moderating effect of organizational identification. Results reveal that voluntary telecommuting leads to more emotional support and innovative behavior than involuntary telecommuting, and organizational identification enlarges the difference in emotional support [136].

Well-being (cluster #5) was a central topic of discussion, especially during and after the pandemic. Other related topics such as mental health (cluster #10), employee well-being (cluster #1–5), job satisfaction (cluster #1–8), and loneliness (cluster #3–15) were also identified through our analysis. Golden addressed the inconsistency in studies that investigated the relationship between telecommuting and job satisfaction, arguing that the relationship is curvilinear with extent telecommuting, moderated by task interdependence and job discretion, which plateau at more extensive levels [137]. Additionally, relationship aspects such as leader-member exchange quality, team-member exchange quality, and work-family conflict can play a mediation role [138]. Golden also found that teleworking has a positive association with organizational commitment and a negative one with turnover intentions; however, this relationship is mediated by work exhaustion [139]. On the other hand, teleworkers can feel some degrees of psychological detachment from work, which negatively influences well-being (measured by job satisfaction and emotional exhaustion); however, its impact can be reduced by family interfering with work [140]. Teleworking also has some impact on the mental and physical health of employees. Oakman et al. reviewed the studies on this issue published between 2007 and May 2020 and found that the impact of teleworking on mental and physical health is influenced by the degree of organizational support, social connectedness, colleague support, and work to family conflict [43]. Xiao, YJ et al. investigated the same issue empirically and found that the decreased physical and mental well-being during work from home (WFH) due to COVID-19 was associated with factors such as physical exercise, food intake, communication with coworkers, distractions while working, adjusted work hours, workstation set-up, and satisfaction with workspace indoor environmental factors [42].

### Unintended consequences

The adoption of telework during and after the COVID-19 pandemic has some public (cluster #2–11) and regional (cluster #3–7) implications. Besides, it has some consequences for travel, transportation, and traffic (cluster #4). for public and private organizations, it accelerated and increased the need for digital transformation (cluster #3–14) with some concerns regarding cybersecurity issues (cluster #7).

Rocha et al., analyzed the behavior of production and public spending variables in Brazil between March and July 2020 where 93.2% of the workers adhered to teleworking, using a database extracted from institutional records [141]. The study found that production levels maintained an average behavior after the initial adaptation period, and the number of employees remained stable. Besides, there was a reduction in spending on water, electricity, and travel compared to historical values suggesting that robust planning to rationalize the use of physical environments and structures could have reduced public expenditure in other areas. Office spaces (especially in the center of cities and central business districts) have been less occupied, and their price per square meter has declined [142]. One study estimated a 45% decline in value (in the short term) of New York City office spaces and about 39% decline in the long term [143]. Thus, this affects the office space owners and how the cities function since this may lead the cities to either raise taxes or cut services to make up for such decline [143]. The pandemic also caused a shift in demand away from high-density neighborhoods due to reduced need for proximity to telework-compatible jobs and generally a significant but smaller shift away from large cities [144].

Although urban planners and policymakers have been proposing telecommuting as one of the ways to reduce congestion [145, 146], they should carefully consider other unintended consequences driven by the fast adoption of teleworking. Empirical studies argued that telecommuting has a substitution effect on commute and that it has been an essential factor influencing travel patterns [147–149]. Since it has also been found to reduce total daily vehicle miles traveled [150], it impacts the public transportation and the amount of commuting activities. If this decline in ridership is not recovered, as suggested by some studies that investigated this scenario [151], this will affect the financial side of cities’ transportation systems. Additionally, The impact of teleworking on travel and transportation have consequences for climate and energy [40]. A recent study argues that while teleworking is often seen as more sustainable due to reduced transportation and office space dependency, the paper shows that the situation is more complex than previously thought; pointing out that while some studies claim that teleworking can lower energy consumption, others claim that it may increase it, especially in the transportation sector [66]. The article concludes that current datasets and research methods are insufficient to answer the research question fully. By producing the sub-cluster from cluster #4 we can see that the researchers investigated related topics such as travel patterns and activities (cluster #4–1) and counter urbanization (cluster #4–9) which can be considered emerging topics judging by their average publication year.

**Fig 8:**
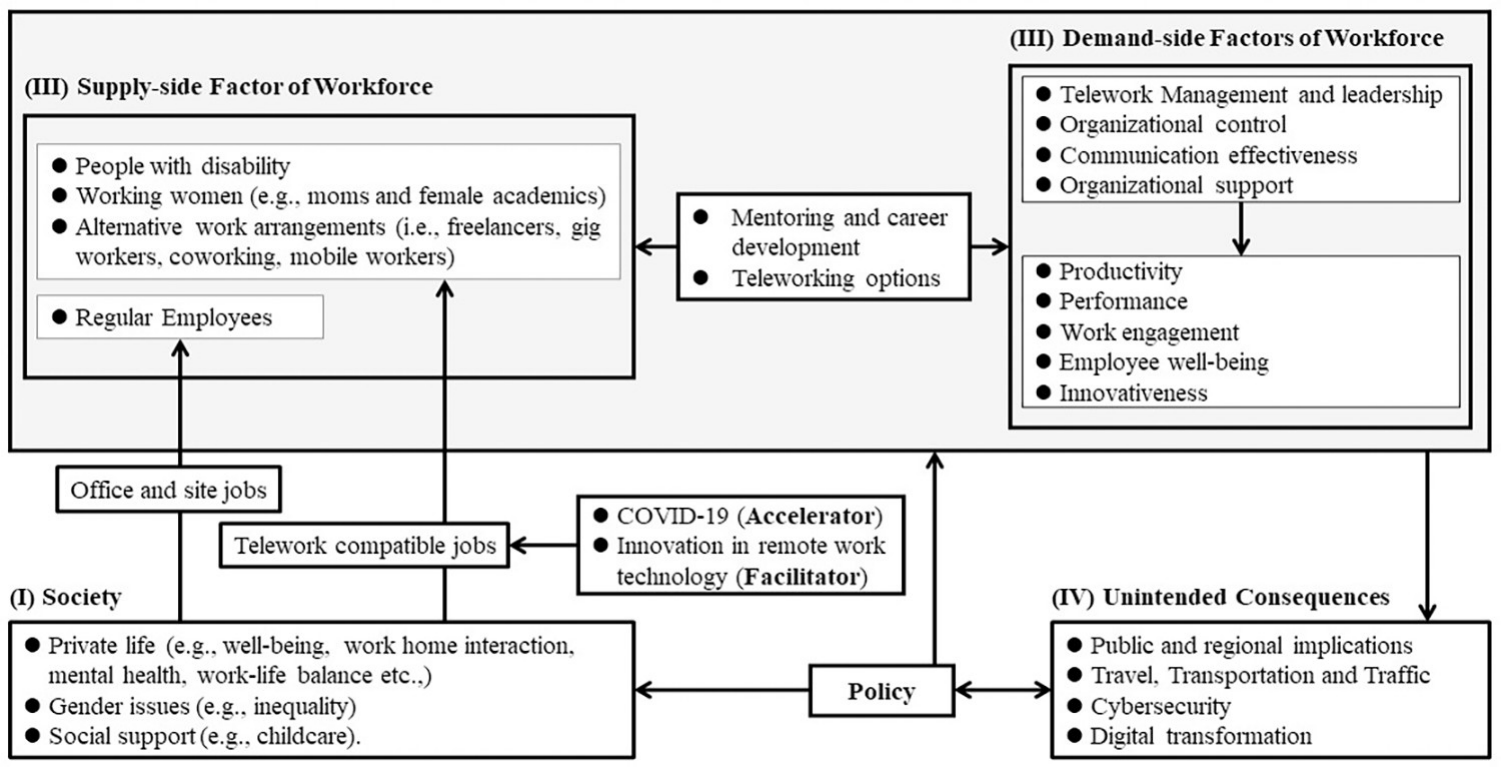
Illustration of how the adoption of teleworking may have influenced the dynamic of labor market.

Regarding organizations, our analysis uncovered two unintended consequences related to the use of technology that have been utilized to facilitate teleworking: cybersecurity (cluster #7) and digital transformation (cluster #3–14). Organizations have had to implement digital tools and technologies, such as video conferencing and collaboration software, at an unprecedented scale to maintain business continuity which led to a faster and more widespread implementation of digital transformation initiatives [152]. However, the shift to remote work has also raised concerns about cybersecurity, where protecting information systems from unauthorized access remains a challenge [73]. Due to less exposure to situational support, verbal persuasion, and vicarious experiences, telecommuters tend to have lower awareness of information security policies [153]. Georgiadou, et al. found significant variations among the organizations that he surveyed in terms of their level of cybersecurity culture and emphasizes the need for continuous training and awareness programs to cultivate cybersecurity culture and the lack of security guidelines and measures provided to teleworking employees during the pandemic [154]. Fig 8 shows an illustration tempting to frame the labor market and workforce dynamics influenced by the rapid teleworking adoption during and after the COVID-19 pandemic.

Topic-specific clusters such as teleworking consequences and travel, transportation, and traffic discuss and investigate issues that can be considered vital for the teleworking research field. Judging by the sub-topics of these two clusters, they have covered most of the leading research questions and issues related to teleworking. On the other hand, the adoption of teleworking as a new way of working has influenced issues related to the employment domain that are covered in domain-specific clusters. For example, cluster #2 discusses teleworking benefits and pitfalls regarding its implications for issues such as organization control, people with disabilities, and coworking spaces. These sub-topics are not exclusively telework-related topics but are influenced by teleworking when adopted. We believe that topics in domain-specific clusters will shift and become topic specific when it becomes the center of attention by teleworking scholars. One candidate for such a transition from domain-specific to topic-specific can be employee well-being (cluster #5). As indicated by the cluster trend in Table 1, the focus on employee well-being by teleworking researchers increased rapidly after the COVID-19 pandemic (the past three years). Moreover, we expect that research on employee well-being will continue to grow as teleworking or working from home is increasingly becoming the new normal.

**Fig 9:**
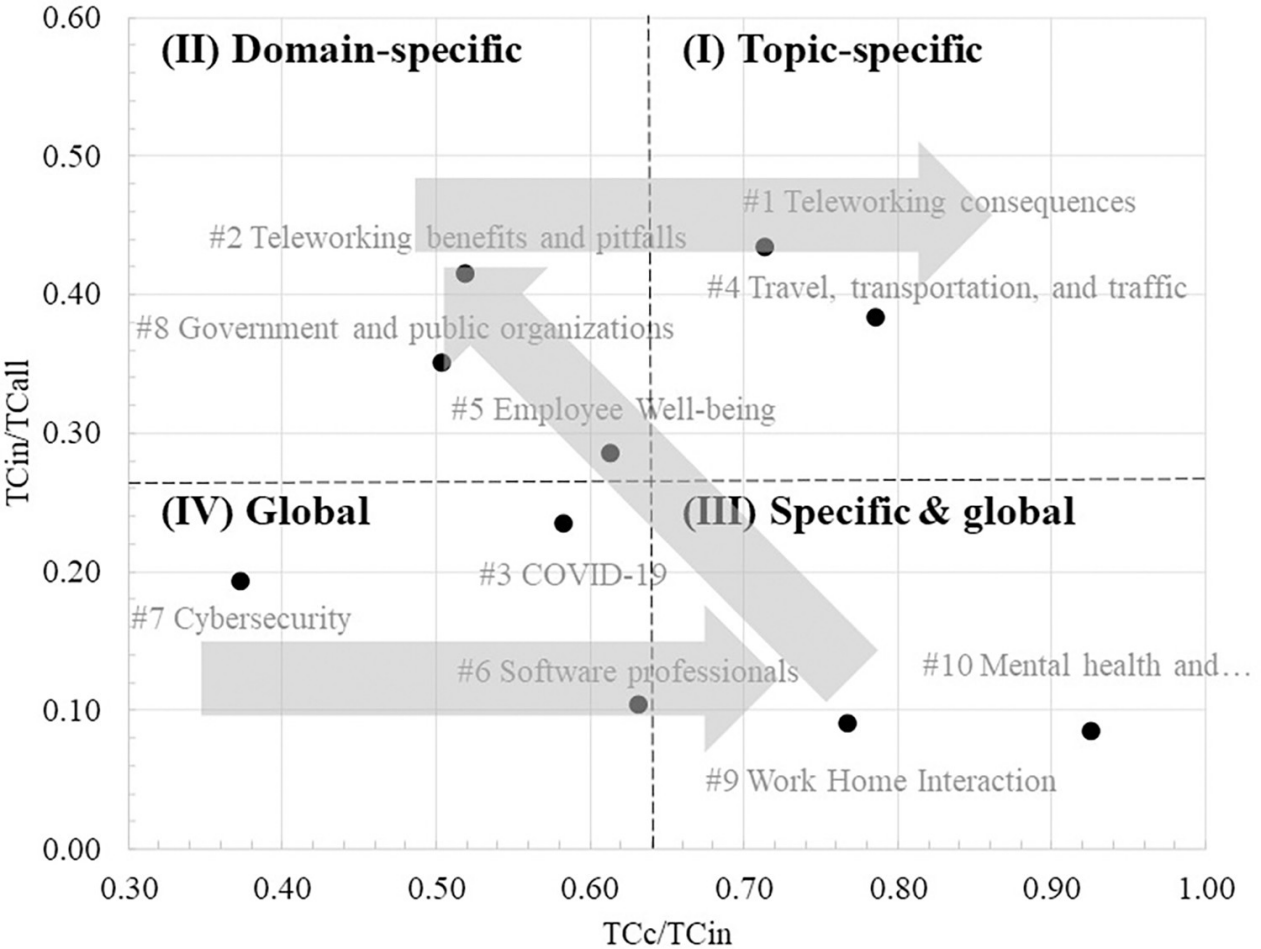
Groups of teleworking research topic’s clusters based on citation relativity analysis and the shift of clusters towards topic-specific group.

Topics in Specific & global cluster group can be thought of as contextual topics or issues that have general impact on employees and at the same time some specific impact on the teleworking employees. For example, work home interaction (cluster #9) discusses sub-topics such as work-home interference and conflict, which become critical concerns during the COVID-19 lockdown, where people are forced to work from home, sharing the same space with children and other family members. The same can be stated regarding cluster #10 (Mental health and psychotherapy), where providers of mental health and psychotherapy services become in high demand during and after the pandemic [56]. Finally, Topics in global clusters, where the articles in these clusters have been cited more by articles outside their cluster and more articles outside our dataset, can be considered as topics newly becoming the subject of discussion within teleworking literature, as can be seen in cluster #3 (COVID-19) and cluster #7 (cybersecurity). The topics in such clusters and another group (such as specific & global or domain-specific) tend to shift towards topic-specific clusters (center of attention) as the discussion about these topics proliferates more through time, raising more research questions and concerns that need to be addressed by teleworking scholars (Fig 9). The same logic can be generalized for the sub-cluster level shown in Fig 7.

## Conclusion and research implications

This study presented a holistic topical overview of the teleworking literature presenting the most important qualitative data by utilizing bibliometric methods which have proven to be useful when dealing with large number of publications. Moreover, based on the synthesis of teleworking research topics and results from our analysis, we proposed a framework attempting to explain and discuss how teleworking adoption during and after COVID-19 may have influenced the labor market and workforce dynamics. We argue that the teleworking adoption have been accelerated by COVID-19 pandemic and facilitated by the innovation in teleworking technology and that individuals join the workforce either through regular office jobs or other working arrangements including teleworking, freelancing, and gig working. We also argue that other flexible working arrangements have increased the labor pool by allowing people with different situations, such as people with disabilities and working mothers, to participate. The lack of support (such as childcare for working mothers or employment opportunities accessible to people with disabilities) may prevent their partial or full participation; thus, policymakers should work on policies that can provide the needed support and resolve any issues that may hinder workforce participation.

The dynamic between the supply (employees) and demand (organizations) sides and the decision of an employee to join a particular organization are influenced by some factors such as the availability of teleworking options (full or hybrid) and mentoring and career growth opportunities. On the other hand, organizational control, telework management and leadership, organizational support, and communication effectiveness are vital for firms as they can influence employees’ outcomes including productivity, performance, work engagement, employee well-being, and innovativeness. Coordination and communication between all stakeholders can be very critical, and for building an effective work policy, organizations need to account for efficiency and personal preference for developing more flexible and sustainable work policies [155]. Mastering of new technologies will increase the teleworking voluntariness [156], and employees need to improve their digital skills to meet the increasing demands for digital literacy to improving workforce resilience [108].

The change in the dynamics between labor supply and demand, beside its effect on travel, transportation and traffic, led to unintended consequences and affect other economic aspects since it has public and regional implications. Other consequences include pushing organizations to think more seriously about their digital transformation initiatives and address their cybersecurity issues which can be vital in such context [73, 152]. Government officials and policy makers may need to change or establish new policies to deal with the negative aspects of these unintended outcomes and regulate the dynamics between the labor supply and demand. In this rapidly changing environment, keeping a fixed policy around teleworking for organizations and the government can be impractical; thus, we may need to be more flexible to enhance organizations’ and society’s resilience, and to maintain sustainable growth [157, 158].

In interpreting the results of teleworking studies conducted during the COVID-19 pandemic, it is important to exercise caution before generalizing these findings to broader teleworking experiences. The unique context of the pandemic, where teleworking was mandated as a response to social distancing measures, introduced specific stressors for employees. These included the direct stress associated with the pandemic and challenges related to isolation and the implementation of social distancing [159, 160]. These factors likely influenced employees’ teleworking experiences during this period, potentially making them unrepresentative of teleworking under normal circumstances. Given this context, it is essential to recognize that the teleworking experiences documented during the COVID-19 pandemic may differ from those in more typical situations. The compounded stressors of the pandemic environment may have skewed perceptions and experiences of teleworking. Therefore, applying the findings from pandemic-related teleworking studies to general teleworking practices should be done with caution and consideration of these contextual differences [35]. This approach can ensures a more accurate and relevant application of these findings to future teleworking policies and practices outside the extraordinary circumstances of a global health crisis.

Based on the trends we observed regarding teleworking and other work arrangements to accommodate workers with various situations and conditions, with proper job design, leadership and organisational support and adequate information communication technology (ICT), teleworking will be central to the future of jobs [161].

### Limitations and future research agenda

In our study, we utilized citation analysis to effectively identify emerging research trends, major topics, sub-topics, and gaps in the field, relying solely on the Web of Science (WoS) database. Despite this focus, it is comparable to other databases like Scopus [162], and Bar-Ilan [163] noted the significant coverage overlap with Scopus, particularly from 1996 onwards, indicating a comparable citation landscape. Moreover, it is frequently used and proven valuable for gaining broad perspectives on research fields and is considered reliable for bibliometric analysis [26]. For example, this statement is supported by Singh et al. [164], recognizing its precision in subject classification, surpassing that of Scopus and Dimensions, which is essential for accurate bibliometric analysis.

Discussions outside the academic channels, such as social media platforms, shows that the COVID-19 pandemic has significantly impacted public perceptions of teleworking and other forms of work arrangements, like gig work; and such data need to be considered for broader understanding of the topic. Studies show varied sentiments: Alotaibi and Alharbi [165] reported neutral to positive views on teleworking in Saudi Arabia, emphasizing flexibility and teamwork, while Zhang, Yu, and Marin [166] found generally positive attitudes towards remote work, focusing on mental health and work-life balance. However, a study analyzing around 11,000 tweets [167] revealed a need for more public awareness of the environmental benefits of teleworking. In the gig economy, a study of 23,845 Twitter posts [168] showed a 150% increase in positive sentiments towards services like ride-hailing and food delivery. This indicates a shift in public attitude, with fewer negative experiences and more support for gig workers, suggesting implications for policy and workforce strategies in these sectors. In this study, our methodology was primarily centered on analyzing academic articles. However, since teleworking is widely discussed outside of traditional academic channels, future research can explore new methodologies to integrate this additional information to obtain more extensive view.

In this review, we outlined the most central topics of teleworking research, and in doing so, some topics appeared more salient (i.e., COVID-19, well-being, travel and transportation, and technology related issues). We suggest that researchers pay more attention to the role of technology by investigating how innovations in remote work technologies, such as virtual reality (VR), augmented reality (AR), and metaverse platforms, could enhance the teleworking experience and reduce its pitfalls. Besides, we should look carefully into how such technologies may impact employees to alleviate some of the social and psychological problems experienced by teleworkers and their shadows on families and society. Compared to the attention devoted to the role of innovation in teleworking technologies, telework scholars have yet to give much attention to employees’ innovativeness (cluster #1–12). Our analysis showed a very limited number of publications related to this topic (8 articles). Judging by the average publication year of this sub-cluster (2021.7), this sub-topic can be considered an emerging research topic. Future work should attempt to fill this research gap and propose an effective and sustainable work model that could benefit both employees and employers by supporting and enhancing employees’ innovativeness for better competitive advantages. Finally, teleworking researchers should put more focus on other working arrangements such as gig workers, freelancers, and mobile teleworkers. These working arrangements remains under-investigated, as our literature review revealed a limited number of studies.
